# LncRNA H19 is a major mediator of doxorubicin chemoresistance in breast cancer cells through a cullin4A-MDR1 pathway

**DOI:** 10.18632/oncotarget.21121

**Published:** 2017-09-21

**Authors:** Qiong-Ni Zhu, Guo Wang, Ying Guo, Yan Peng, Rui Zhang, Jun-Li Deng, Zhi-Xing Li, Yuan-Shan Zhu

**Affiliations:** ^1^ Department of Clinical Pharmacology, Xiangya Hospital, Institute of Clinical Pharmacology, Central South University, Changsha, P. R. China; ^2^ Department of Medicine, Weill Cornell Medical College, New York, NY, USA

**Keywords:** chemoresistance, lncRNA H19, CUL4A, ABCB1, breast cancer

## Abstract

Development of chemoresistance is a persistent problem during cancer treatment. Long non-coding RNAs (LncRNAs) are currently emerging as an integral functional component of the human genome and as critical regulators of cancer development and progression. In the present study, we investigated the role and molecular mechanism of H19 lncRNA in chemoresistance development by using doxorubicin (Dox) resistance in breast cancer cells as a model system. *H19* lncRNA expression was significantly increased in anthracycline-treated and Dox-resistant MCF-7 breast cancer cells. This H19 overexpression was contributed to cancer cell resistance to anthracyclines and paclitaxel as knockdown of *H19* lncRNA by a specific *H19* shRNA in Dox-resistant cells significantly improved the cell sensitivity to anthracyclines and paclitaxel. Furthermore, gene expression profiling analysis revealed that a total of 192 genes were associated with H19-mediated Dox resistance. These genes were enriched in multiple KEGG pathways that are related to chemoresistance. Using genetic and pharmacological approaches, we demonstrated that MDR1 and MRP4 were major effectors of H19-regulated Dox resistance in breast cancer cells as MDR1 and MRP4 expression was markedly elevated in Dox-resistant cells while dramatically reduced when H19 was knocked down. Moreover, we found that CUL4A, an ubiquitin ligase component, was a critical factor bridging H19 lncRNA to MDR1 expression, and a high tumor CUL4A expression was associated with low survival in breast cancer patients treated with chemotherapy. These data suggest that H19 lncRNA plays a leading role in breast cancer chemoresistance, mediated mainly through a *H19*-*CUL4A*-*ABCB1*/*MDR1* pathway.

## INTRODUCTION

Breast cancer is the most common malignancy in women in the United States and is second only to lung cancer as a cause of cancer death [1]. In China, an increasing trend in mortality is observed for 3 of the 10 most common cancers (breast, cervix, and ovary), while it tends to be stable for others such as colorectal, lung, uterine, and thyroid cancers [2, 3]. Breast cancer accounts for approximately one million new cases and leads to more than 400,000 deaths per year in the world. Chemotherapy is one of the basic treatments for cancers [4]. With the advent of various anticancer drugs, cytotoxic and molecularly targeted compounds have become the first-line standard treatment regimens for most cancer patients when surgery is not an appropriate option [5, 6]. However, drug resistance is a major reason for the failure of clinical treatment. The combination of anthracycline antibiotics such as doxorubicin and cyclophosphamide is one of the most basic and important parts in the recommended first-line chemotherapy of breast cancer [1]. About 30% of clinical patients under systemic chemotherapy for breast cancer develop multidrug resistance (MDR) and relapse with a worse prognosis due to drug resistance [7]. Overall, there is a high incidence of chemoresistance with a poor prognosis in breast cancer.

The dysfunction of long non-coding RNAs (lncRNAs) has been implicated in various human diseases [8]. Recently, growing evidence has indicated that lncRNA expression is widely altered in cancers, and lncRNAs are associated with various aspects of tumorigenesis through inactivation of tumor suppressors or activation of oncogenes [9–11]. LncRNA-*ATB* has been shown to be remarkably upregulated in trastuzumab-resistant breast cancer cells and tissues and to promote trastuzumab resistance [12]. Jiang et al. have reported that lncRNA *HIF1A*-*AS2* and *AK124454* contributed to paclitaxel resistance in triple-negative breast cancer through transcriptome analysis [13]. Moreover, lncRNA-*ARA* has been shown to promote doxorubicin resistance in breast cancer cells [14]. These data collectively suggest the involvement of lncRNAs in breast cancer chemoresistance, and the identification of new lncRNAs that are linked to breast cancer chemoresistance is imperative for the understanding of chemoresistance development and the discovery of new therapeutic targets for chemoresistant breast cancer patients [4, 15].

The imprinted oncofetal *H19* gene is expressed in the embryo, down-regulated at birth and then overexpressed in tumors. Its role in tumor initiation and progression has long been a subject of controversy, although accumulating data suggest that H19 is a major gene involved in cancer [16–18]. *H19* lncRNA is highly expressed in a variety of human cancers, including breast cancer, colorectal cancer, hepatocellular carcinoma, and gastric cancer [19–22], and its overexpression is often correlated with poor prognosis in cancer patients [23–25]. Particularly, *H19* lncRNA is overexpressed in approximately 70% of breast cancers [2]. However, the functional significance and mechanism of *H19* lncRNA in breast cancer chemoresistance is poorly understood despite its significant role in cancer development and progression.

In the present study, we determined the importance of *H19* lncRNA in breast cancer chemoresistance using doxorubicin as a model chemotherapeutic agent, and revealed for the first time that H19 lncRNA plays a leading role in breast cancer chemoresistance development, mediated mainly through a *H19*-*CUL4A*-*ABCB1*/MDR1 pathway.

## RESULTS

### The expression of H19 lncRNA is increased in Dox-treated and Dox-resistant MCF-7 breast cancer cells

**Figure 1:**
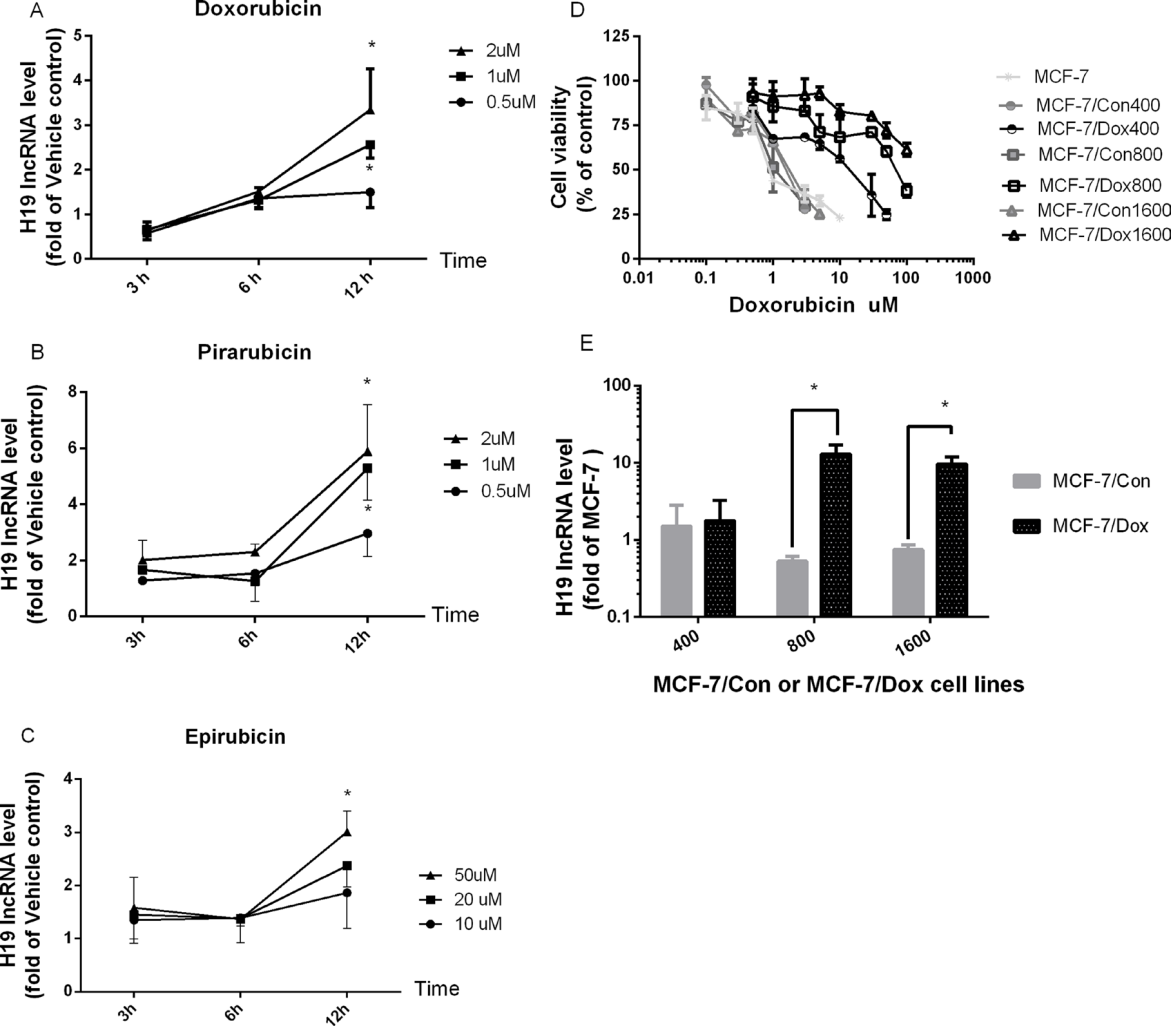
The expression of H19 lncRNA is increased in Dox-treated and Dox-resistant MCF-7 breast cancer cells MCF-7 cells were treated with vehicle control or various doses of doxorubicin (**A**), pirarubicin (**B**) and epirubicin (**C**) for 3, 6 and 12 hours (h), respectively, and total cellular RNAs were extracted from harvested cells at the end of treatment. H19 lncRNA level was quantified by real-time RT-PCR, normalized to internal control and expressed as fold of corresponding vehicle control. The data in panels A–C are the mean ± SEM of three independent experiments. ^*^p < 0.05 compared to corresponding controls. In panel D, dose-response analyses of doxorubicin effect on inhibition of viable cell numbers (cell viability) were carried out in parental MCF-7, Dox-resistant (MCF-7/Dox400, MCF-7/Dox800 and MCF-7/Dox1600) and the parallel control cells (MCF-7/Con400, MCF-7/Con800 & MCF-7/Con1600). Cells were plated in 96-well plates and treated with various doses of doxorubicin for 48 h. The viable cell number was expressed as percentage of corresponding vehicle controls and the values are the mean ± SEM of two or three independent triplicate experiments. In panel E, the levels of H19 lncRNA were determined by real-time RT-PCR in parental MCF-7, Dox-resistant (MCF-7/Dox) and the parallel control cells (MCF-7/Con), and expressed as fold of parental MCF-7 control. The values are the mean ± SEM of two to five independent experiments. ^*^p < 0.05 compared to parallel control and parental MCF-7 group.

To determine if *H19* lncRNA expression is influenced by anthracycline drugs, we first examined the effects of anthracycline drugs doxorubicin (Dox), epirubicin and pirarubicin on H19 lncRNA expression in MCF-7 cells. As shown in Figure 1A, Dox produced a time- and dose-dependent induction of H19 lncRNA expression, and its expression was significantly increased at 1 and 2 μM of Dox when treated for 12 h. At 2 μM, Dox induced H19 expression more than 3.5 fold. Similar time- and dose-dependent induction of H19 lncRNA expression was observed for pirarubicin (Figure 1B) and epirubicin (Figure 1C) in MCF-7 cells. Moreover, the expression of H19 lncRNA increased more than 15 fold in Dox-resistant cells, MCF-7/Dox800 and MCF-7/Dox1600, compared to parental MCF-7 cells (Figure 1E). The increase in H19 expression in Dox-resistant cells was associated with an increase in chemoresistance as demonstrated by the IC_50_ of Dox in cell viability as shown in Figure 1D and Table 1. The IC_50_ of Dox was approximately 0.8 μM in parental MCF-7 cells, while it was 21.1, 79.9 and 270 μM in Dox-resistant MCF-7/Dox400, MCF-7/Dox800 and MCF-7/Dox1600 cells (Figure 1D and Table 1), respectively.

**Table 1:** The IC50 values (μM) of various chemotherapeutic agents on cell viability in breast cancer cell lines

Cells	Doxorubicin	Pirarubicin	Epirubicin	Paclitaxel	CDDP
**MCF-7/Con1600**	1.81 ± 0.45	0.50 ± 0.08	10.00 ± 0.42	1.95 ± 0.56	7.98 ± 3.86
**MCF-7/Dox1600**	270.00 ± 46.00^*^	19.19 ± 7.58^*^	228.24 ± 127.66^*^	294.23 ± 178.02^*^	21.76 ± 4.89^*^
**MCF-7/Dox/NC**	302 ± 87.91^*^	20.90 ± 2.31^*^	175.55 ± 105.25^*^	278.01 ± 46.43^*^	18.66 ± 6.06^*^
**MCF-7/Dox/shH19**	26.32 ± 1.81^#^*^&^	3.44 ± 1.63^#^*^&^	32.65 ± 7.54^#^*^&^	9.38 ± 3.36^#^*^&^	19.38 ± 4.65^*^

^*^p < 0.05 compared to MCF-7/Con1600 group; ^#^*p* < 0.05 compared to MCF-7/Dox/NC group; ^&^*p* < 0.05 compared to MCF-7/Dox1600 group.

### Knockdown of H19 lncRNA reverses chemoresistance in Dox-resistant breast cancer cells

**Figure 2:**
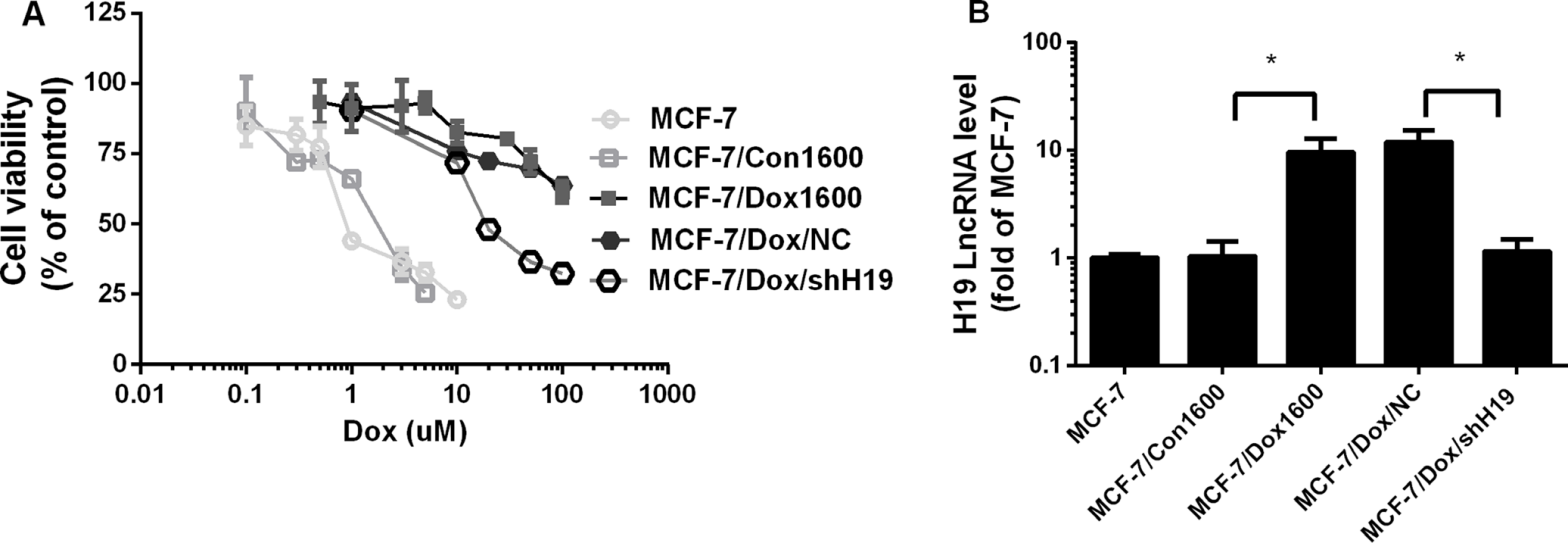
Knockdown of H19 lncRNA reverses chemoresistance in Dox-resistant breast cancer cells In panel **A**, MCF-7 control, Dox-resistant MCF-7/Dox1600 and H19-knockdown MCF-7/Dox/shH19 cells were plated in 96-well plates and treated with various doses of Dox for 48 h. The number of viable cells (cell viability) was determined at the end of treatment and expressed as a percentage of corresponding vehicle controls. The data are the mean ± SEM of two or three independent triplicate experiments. In panel **B** the levels of H19 lncRNA were quantified using real-time RT-PCR in Dox-resistant MCF-7/Dox1600, H19-knockdown MCF-7/Dox/shH19 and the corresponding control cells and expressed as fold of parental MCF-7 control. The data are the mean ± SEM of three independen*t* tests. ^*^p < 0.05 compared to corresponding controls and parental MCF-7 cells.

To determine the contribution of lncRNA *H19* to chemoresistance in breast cancer cells, a cell line, MCF-7/Dox1600/shH19, was established by stable transfection of a specific H19 shRNA expression vector in MCF-7/Dox1600 cells to knock down *H19* expression as described in the Materials and Methods. Compared to the original MCF-7/Dox1600 cells, the expression of *H19* lncRNA was knocked down more than 90% in the MCF-7/Dox1600/shH19 cells using real-time RT-PCR analysis (Figure 2B). However, H19 expression was not affected in the control vector transfected MCF-7/Dox1600/NC cells. When H19 lncRNA was knocked down, the Dox-resistant cells were much more sensitive to Dox therapy with a dramatical decrease in IC_50_ of cell viability from 270 μM in the original MCF-7/Dox1600 cells to 26.23 μM in the H19 knockdown MCF-7/Dox/shH19 cells (Figure 2A and Table 1). Very interestingly, Dox-resistant MCF-7/Dox1600 cells were also resistant to other chemotherapeutic agents including epirubicin, pirarubicin, paclitaxel and CDDP (Table 1). When H19 lncRNA was knocked down in MCF-7/Dox1600 cells, cell sensitivity to epirubicin, pirarubicin and paclitaxel, but not CDDP, was significantly restored as evident by a significant decrease in its IC_50_ value of cell viability (Table 1).

### Alternation in H19 lncRNA level is associated with changes in various gene expression and signaling pathways in Dox-resistant cells

**Figure 3:**
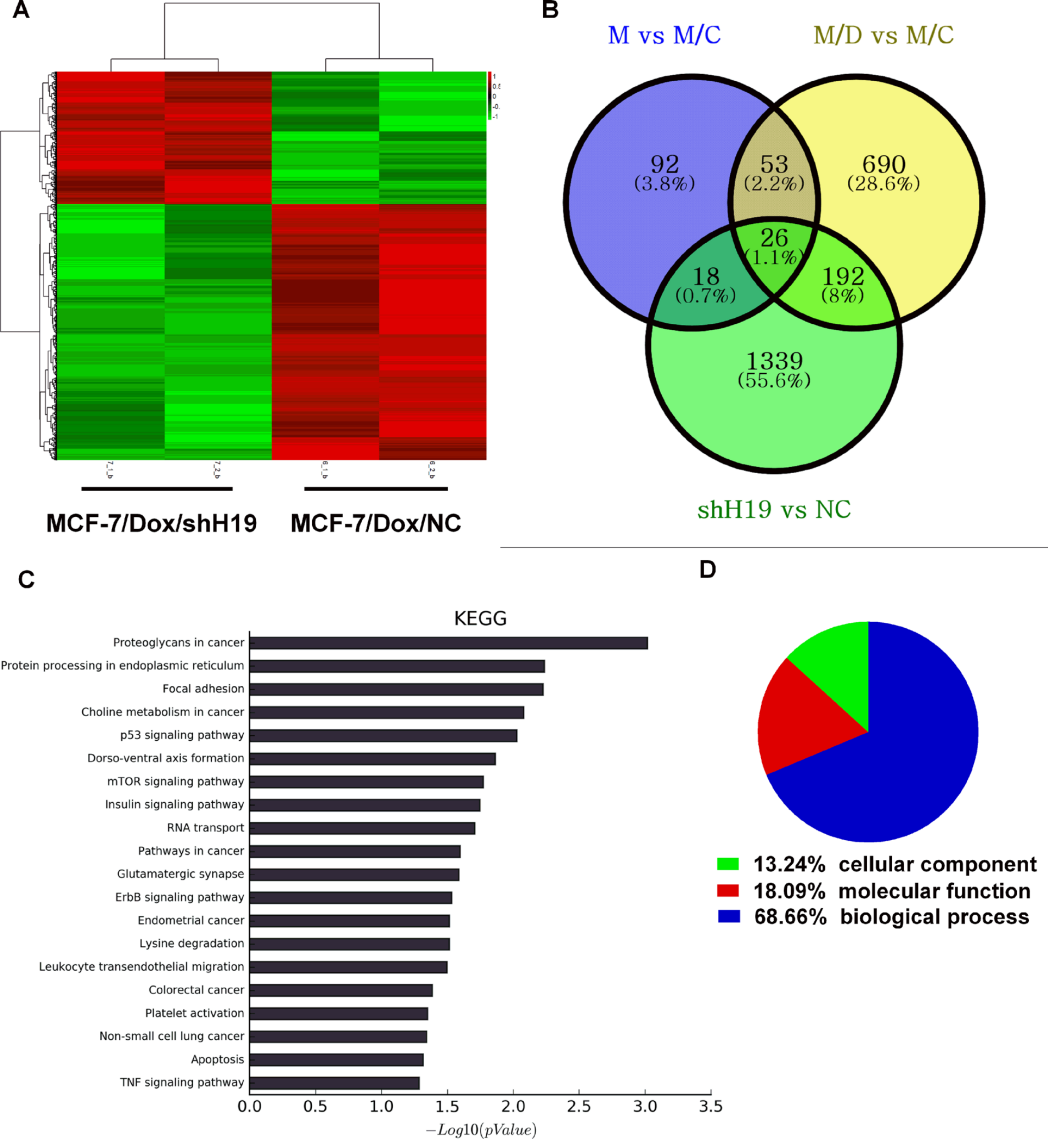
Alternation in H19 lncRNA expression is associated with differential changes in gene expression profiles and signaling pathways in Dox-resistant cells Panel **A** shows a heat map from hierarchical clustering of differentially expressed genes in H19-knockdown MCF-7/Dox/shH19 cells versus the control MCF-7/Dox/NC cells. The red-color represents up-regulated genes and the green color represents the down-regulated genes. Each row represents a single gene. Panel **B** is a Venn diagram depicting the commonly and differentially expressed genes among control, Dox-resistant and H19-knockdown Dox-resistant cells. After overlapping, a total of 192 genes were identified to be associated with H19-mediated chemoresistance. The blue circle represents the differentially expressed genes between parental MCF-7 (M) and parallel control MCF-7/Con1600 (M/C) cells, the yellow circle represents differentially expressed genes between the Dox-resistant MCF-7/Dox1600 (M/D) and the parallel control M/C, and the green circle represents differentially expressed genes between H19 knockdown MCF-7/Dox/shH19 (shH19) and the control MCF-7/Dox/NC (NC) cells. Panel **C** shows the KEGG analysis of the top 20 significantly altered pathways upon H19 knockdown in Dox-resistant cells. *P* values < 0.05 and false discovery rates < 0.05 were used as thresholds to select significant KEGG pathways. The horizontal axis, -log10(pValue), denotes the significance of specific pathways in H19-knockdown MCF7/Dox/H19 cells compared to the corresponding control MCF-7/Dox/NC cells. Panel **D** shows the GO analysis of the fraction of differentially expressed genes in H19-knockdown MCF7/Dox/shH19 versus the control MCF-7/Dox/NC cells in three GO classifications of cellular component, biological process and molecular function.

To gain further insight into the functional roles of *H19* in acquired doxorubicin resistance and other biological functions, mRNA expression profiling in parental MCF-7, MCF-7/Con1600, MCF-7/Dox1600, and H19 knockdown MCF-7/Dox/shH19 cells were determined by microarray. We identified 961 mRNAs that were significantly differentially expressed (*P* < 0.05; fold change > 2) between MCF-7/Dox1600 and MCF-7/Con1600 (Figure 3B). After knockdown of *H19* expression in MCF-7/Dox1600 cells, 1575 mRNAs that were significantly differentially expressed, 1004 mRNAs were down-regulated while 571 were up-regulated in the H19 knockdown MCF-7/Dox1600/shH19 cells (Figure 3B). Hierarchical clustering was performed to represent the differential mRNA expression profiles (Figure 3A). Overlapping genes differentially expressed in MCF-7/Dox1600 and MCF-7/Dox1600/shH19 cells indicated that a total of 192 differentially expressed genes (DEGs) were probably related to Dox chemoresistance (Figure 3B and Supplementary Table 1).

To investigate the functional significance of H19-regulated genes, pathway-expression analysis was performed and genes regulated by *H19* were significantly enriched in multiple KEGG pathways, including protein processing in endoplasmic reticulum, focal adhesion, p53 signaling pathway, mTOR signaling pathway, etc. as shown in Figure 3C. Most of those pathways are strongly associated with chemoresistance based on previous reports as shown in Table 2. GO analysis indicated that 69% of these differentially expressed genes were related to biological processing, 18% to molecular functions, and 13% to cell components (Figure 3D).

**Table 2:** Pathways strongly associated with chemoresistance as previously reported

Pathways	PMIDs of Reports
**RNA transport**	**24740415**
**mTOR signaling pathway**	**27765907**
**Insulin signaling pathway**	**24885964, 22275271**
**Platelet activation**	**24885964, 22275271**
**p53 signaling pathway**	**27807478**
**Protein processing in endoplasmic reticulum**	**21899885**
**Choline metabolism in cancer**	**27446799**
**FoxO signaling pathway**	**18390843**
**Endocytosis**	**27613838**
**RNA degradation**	**26751936, 24368600**

### Doxorubicin chemoresistance is associated to H19-mediated upregulation of multidrug resistant proteins (MDR)

**Figure 4:**
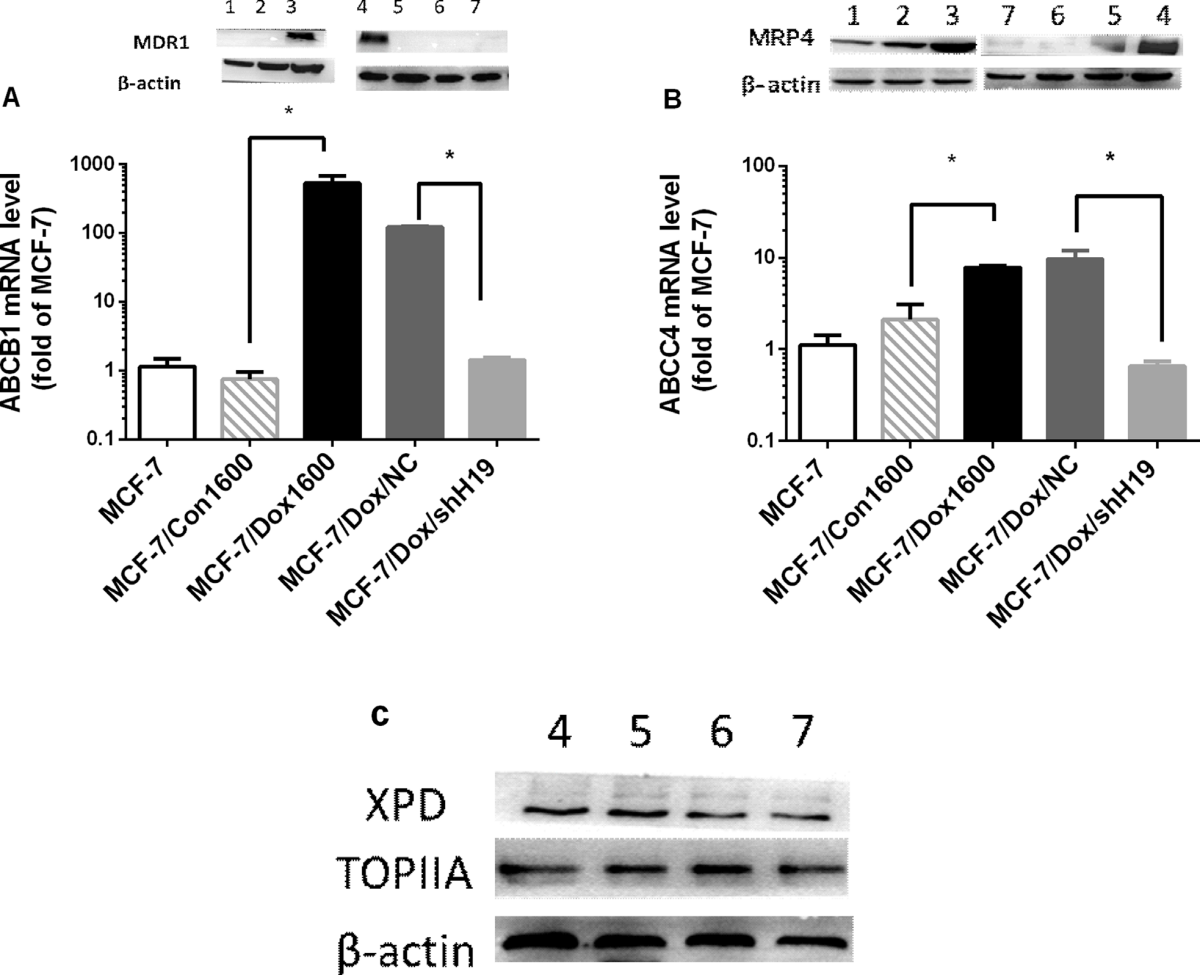
Doxorubicin chemoresistance is associated to H19-mediated upregulation of multidrug resistant proteins (MDR) In panels **A** and **B**, the levels of *ABCB1* and *ABCC4* mRNAs, and MDR1 and MRP4 proteins were quantified respectively by real-time RT-PCR and Western blot analysis in various cell lines as indicated. The mRNA levels were expressed as fold of parental MCF-7 control, and the data are the mean ± SEM of three to five independent experiments. The inserts on top of panels A and B, and panel **C** are representative Western blots analyses of MDR1, MRP4, XPD and TOPIIA proteins, respectively. β-actin was used as an internal loading control in Western blot analysis. Lanes in all Western blots are denoted as: lane 1 – MCF-7, lane 2 – MCF-7/Con1600, lane 3 – MCF-7/Dox1600, lane 4 – MCF-7/Dox/NC and lanes 5 to 7 – Three independent MCF-7/Dox/shH19 clones. ^*^p < 0.05 compared to the corresponding controls.

Based on the gene profile analysis, we selected several H19-regulated and chemoresistance-associated genes for further analysis. *ABCB1*, a member of the ATP-binding cassette family, which encodes MDR1, a key molecule in multidrug disposition and multidrug resistance, was dramatically upregulated for approximately 529 folds in mRNA level in MCF-7/Dox1600 cells compared to the parallel control cells and greatly decreased when *H19* was knocked down in MCF-7/Dox1600/shH19 cells (Figure 4A). Consistent with *ABCB1* mRNA changes, the level of MDR1 protein was markedly elevated in Dox-resistant cells, and returned to undetectable level in *H19* knockdown cells (Figure 4A inserts). Furthermore, the expression of another ATP-binding cassette sub-family member, *ABCC4* that encodes the multidrug resistance-associated protein 4 (MRP4) was also significantly upregulated in Dox-resistant cells and backed to control level when *H19* was knocked down using both quantitative RT-PCR and Western blot analysis (Figure 4B). In contrast, the levels of XPD and TOPIIA proteins, two previously reported molecules related to doxorubicin sensitivity [26] were not significantly altered in Dox-resistant cells or *H19* knockdown cells (Figure 4C).

### CUL4A is a key factor in H19-mediated chemoresistance and in breast cancer survival

**Figure 5:**
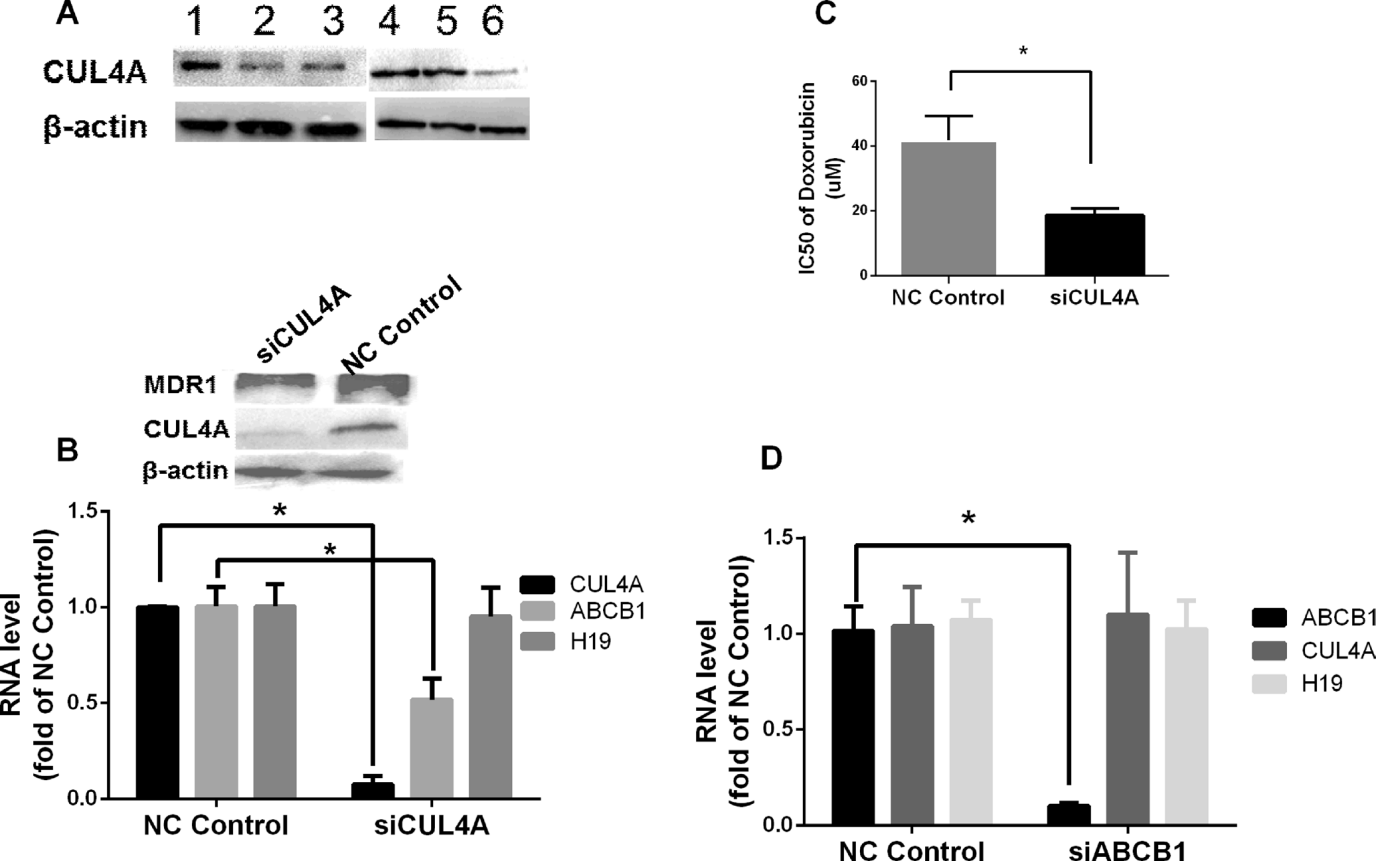
CUL4A is a key H19 downstream factor in H19-mediated chemoresistance in breast cancer cells In panel **A** representative Western blots are shown to illustrate an increase in CUL4A level in Dox-resistant MCF-7/Dox1600 cells (lanes 1 & 4). β-actin was used as an internal loading control. Cells used in the Western blots are: lanes 1 & 4 – MCF-7/Dox1600, lane 2 – MCF-7/Con1600, lane 3 – MCF-7, lane 5 – MCF-7/Dox/NC, and lane 6 – MCF-7/Dox/shH19. In panels **B** and **D** the levels of *H19* lncRNA, *CUL4A* and *ABCB1* mRNA were quantified by real-time RT-PCR in Dox-resistant MCF-7/Dox1600 cells with a transit transfection of a negative control siRNA (NC control), a specific *CUL4A* siRNA (siCUL4A, 100 nM in panel B) or a specific *ABCB1* siRNA (siABCB1, 100 nM in panel D) for 72 h. The RNA levels were expressed as fold of corresponding NC control and the values are the mean ± SEM of three independent experiments. ^*^p < 0.05 compared to the corresponding NC control. In panel **C** Dox-resistant MCF-7/Dox1600 cells were transiently transfected with either a negative control siRNA (NC control) or a specific *CUL4A* siRNA (siCUL4A) at 100 nM concentration. Twenty-four hours after transfection, the cells were treated with either a vehicle control or various doses of doxorubicin for 48 h. Cell viability was determined at the end of experiments and IC_50_ was deducted from the dose-response study as described in the Methods. ^*^p < 0.05.

As CUL4A, an ubiquitin ligase component that is involved in ubiquitination of target proteins, has been shown to play a role in MDR1-mediated chemoresistance [27, 28], we studied the bridging function of CUL4A in H19-MDR1 mediated Dox chemoresistance. Compared to the parental MCF-7 and parallel control cells, the level of CUL4A protein was significantly increased in Dox-resistant MCF-7/Dox1600 cells, and it was greatly decreased by knockdown of H19 lncRNA in MCF-7/Dox1600/shH19 cells (Figure 5A). To explore the functional significance of CUL4A and its relationship with *ABCB1* gene, we specifically knocked down *CUL4A* expression in Dox-resistant MCF-7/Dox1600 cells using siRNA. As shown in Figure 5B, knockdown of *CUL4A* in MCF-7/Dox1600 cells greatly reduced *ABCB1* gene expression as determined at both mRNA and protein levels while it had no effect on lncRNA H19 expression. Consequently, the sensitivity of Dox-resistant cells to Dox was markedly elevated as evident by a dramatic decrease in Dox IC_50_ on cell viability (Figure 5C). On the other hand, knockdown of *ABCB1*, a major effector of Dox chemoresistance, did not affect *CUL4A* and *H19* expression in Dox-resistant MCF-7/Dox1600 cells. In addition, analysis of a dataset in GEO (GSE52544) with *CUL4A* depletion in breast cancer cells indicated that 7 molecular pathways affected by *CUL4A* knockdown were converged to those with *H19* knockdown (Table 3).

**Table 3:** Alterations in pathways that matchup between CUL4A and H19 knockdown in breast cancer cells

BSID	Accession #	Name	Source
921162	hsa04068	FoxO signaling pathway	KEGG
777534	hsa04911	Insulin secretion	KEGG
167325	hsa04141	Protein processing in endoplasmic reticulum	KEGG
102279	hsa04144	Endocytosis	KEGG
83055	hsa04115	p53 signaling pathway	KEGG
83054	hsa04110	Cell cycle	KEGG
1268933	R-HSA-5619084	ABC transporter disorders	REACTOME

BSID-BioSystems database identification.

**Figure 6:**
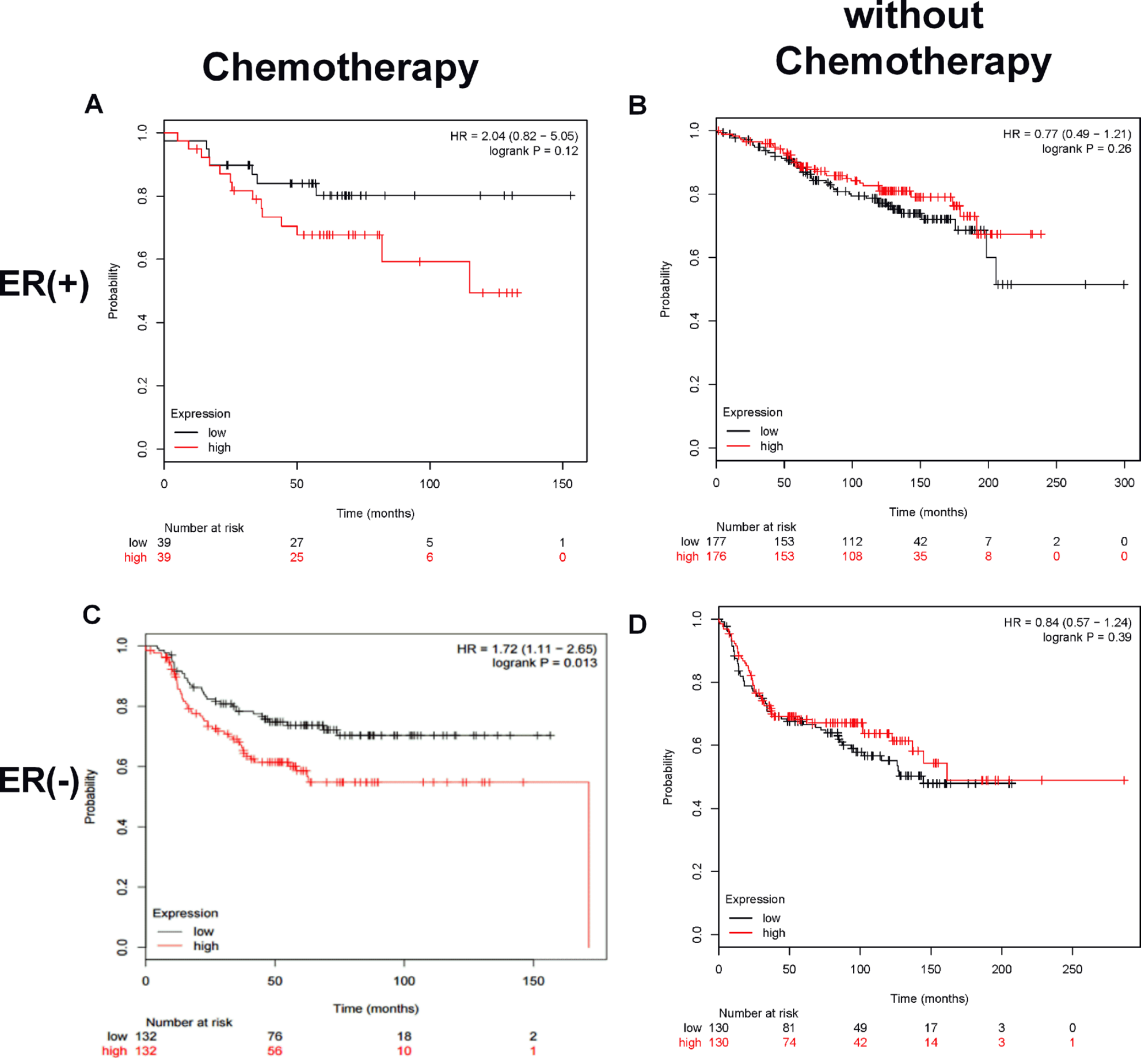
A high tumor CUL4A expression correlates to low survival in breast cancer patients treated with chemotherapy Kaplan–Meier plot analysis of survival curves by tumor *CUL4A* expression levels (low or high), estrogen receptor and chemotherapeutic status was carried out using public datasets as described in the Methods. Panels **A** and **B** are ER^+^ tumors treated with or without chemotherapy, respectively. Panels **C** and **D** are ER^−^ tumors treated with or without chemotherapy, respectively. The red line devotes the high *CUL4A* expression tumors and the black line, low *CUL4A* expression tumors. The hazard ratio (HR) value and log rank *p* value are indicated in each panel.

The importance of CUL4A in breast cancer chemoresistance was further evaluated by analyzing the association of *CUL4A* expression levels in breast cancer tissues to patients’ outcomes in public datasets. Kaplan-Meier survival plot revealed that for all estrogen receptor (ER)-negative breast cancer patients treated with chemotherapy, those with a high *CUL4A* expression in tumor tissues had significantly shorter cancer-specific relapse-free survival (RFS) compared to those with a low *CUL4A* expression (HR = 1.72, 95% confidence interval 1.11–2.65, *p* = 0.013) as shown in Figure 6C. A similar trend was observed in ER-positive breast cancer patients treated with chemotherapy although it was not statistically significant in overall survival due to a small sample size (HR = 2.04, 95% confidence interval 0.82–5.06, *p* = 0.12) as shown in Figure 6A. The survival of breast cancer patients without chemotherapy was not related to tumor *CUL4A* expression (Figure 6B and 6D). These data suggest a potential association between high *CUL4A* expression and poor outcome in breast cancer patients treated with chemotherapy.

## DISCUSSION

Despite the advances in cancer therapy, anthracyclines are still the major chemotherapeutic agents in breast cancer chemotherapy, and chemoresistance is still a main obstacle preventing successful breast cancer treatment [5, 6, 29, 30] as 30% of patients under systemic chemotherapy develop drug resistance and relapse [7, 26]. Over the last decades, studies have revealed multiple cellular and molecular pathways related to anthracycline chemoresistance in a variety of tumor cells including breast cancer cells [31]. However, the molecular mechanism of chemoresistance is far from fully understood, and an improved understanding of the molecular basis of chemoresistance will inevitably lead to the clinical consideration of rational drug combination therapy in cancer patients. Recently, lncRNAs have been emerging as critical integral components of gene regulatory networks, which may play a significant role in tumorigenesis and chemoresistance [4, 12–14, 32]. In the present study, we have investigated the role and molecular mechanism of H19 lncRNA in chemoresistance using doxorubicin resistance in breast cancer cells as a model system, and demonstrated for the first time that H19 lncRNA is a key mediator of chemoresistance in breast cancer cells, functioning at the far upstream of the H19-CUL4A-MDR1 molecular pathway.

*H19*, an imprinting oncofetal gene, has been shown to be overexpressed in multiple cancer tissues including breast cancer [32] and be an estrogen-regulated gene [33]. Using Dox-resistant MCF-7 breast cancer cells developed by incremental increases in Dox concentrations, we observed in the current study that *H19* expression was greatly upregulated in Dox-resistant cells as well as in parental MCF-7 cells treated with anthracyclines (Figure 1). This increase in *H19* expression is contributed to cell resistance to anthracyclines and paclitaxel, but not cisplatin as the IC50s of anthracyclines, paclitaxel and cisplatin on cell viability were dramatically elevated in Dox-resistant cells while markedly decreased once *H19* was specifically knocked down for anthracyclines and paclitaxel but not cisplatin (see Figures 1 and 2, and Table 1). These results are consistent with the fact that anthracyclines analogues (doxorubicin, epirubicin, pirarubicin) and paclitaxel are substrates of MDR1 while CDDP, an inorganic platinum complex that inhibits DNA adduct formation as an antitumor drug, is not a MDR1 substrate. Moreover, this data is in agreement with previous reports that H19 lncRNA expression was increased in Dox-resistant liver cancer cells [21], paclitaxel-resistant breast cancer cell [34], cisplatin-resistant lung cancer cells [25], cisplatin-resistant ovarian cancer cells [25, 35], methotrexate-resistant colorectal cancer cells [36] temozolomide-resistant glioma cells [37] and tamoxifen-resistant breast cancer cells (our unpublished observation), which were shown to contribute to the drug resistance. Furthermore, *H19* upregulation is observed under hypoxia-induced stress in liver and bladder cancer cells [38–40]. Based on the observation that H19 is altered in response to cell stress, and contributes to multiple drug resistance in a variety of cancer cells, we speculate that H19 is a potential master switch in chemoresistance or a common sensor in response to various stressors including chemotherapeutic agents.

The molecular mechanisms of H19 promotion of chemoresistance are far from fully understood although multiple molecular pathways have been reported such as ATP-binding cassette (ABC) transporters [4, 21, 37], BIK [34], glutathione metabolism [25, 35] and Wnt/β-catenin [36]. By comparing gene expression profiles among control, Dox-resistant and H19-knockdown Dox-resistant cells, we identified 192 genes that were associated with H19-mediated chemoresistance in the current study (see Figure 3). These genes are enriched in multiple KEGG pathways that are associated with chemoresistance (Figure 3 and Table 2). Among them, we analyzed two members in ABC family, *ABCB1* and *ABCC4* that respectively encode MDR1 and MRP4, and demonstrated that both genes were significantly upregulated at mRNA and protein levels in Dox-resistant cells and returned to control levels when H19 was knocked down (Figure 4). These observations are not surprising as both MDR1 and MRP4 were well documented to be major effectors of multidrug resistance including anthracycline and paclitaxel resistance [29, 41] and H19 lncRNA has been reported to regulate MDR1 expression in other tumor cells [4, 21, 37]. It is worth to note that the levels of XPD and TOPIIA proteins (Figure 4C), two previously reported molecules related to doxorubicin sensitivity [26], and two other ABC transporters, ABCG2 and MRP1 (data not shown) were not significantly altered in Dox-resistant MCF-7/Dox1600 or H19-knockdown cells (Figure 4C), indicating a relative specificity of H19 lncRNA action on gene regulation.

Most interestingly, we have discovered in the present study that CUL4A protein, an ubiquitin ligase component involved in the degradation of DNA damage-response proteins [27], was significantly elevated in Dox-resistant cells yet returned to control level in H19-knockdown cells (Figure 5A). Moreover, *CUL4A* knockdown in Dox-resistant cells significantly decreased *ABCB1* expression at both mRNA and MDR1 protein levels without an alteration in H19 lncRNA level (Figure 5B), leading to a substantial increase in cell sensitivity to Dox as evident by a marked decrease in IC50 of Dox inhibition of cell viability (Figure 5C). These results are in agreement with a previous report that CUL4A was involved in multidrug resistance in breast cancer cells through regulation of MDR1/P-gp expression [28]. The significance of CUL4A in chemoresistance is further exemplified by analyzing public breast cancer datasets, revealing that a high tumor *CUL4A* expression correlates to low survival in breast cancer patients treated with chemotherapy, but not in those without chemotherapy (Figure 6), implying that CUL4A is involved in cell sensitivity to chemotherapeutic agents. Taken together, these data suggest that CUL4A is a critical component in chemoresistance, and *H19*, *CUL4A*, *ABCB1 and ABCC4* work coordinately to display the action on modulation of multidrug resistance in breast cancer cells.

How *H19, CUL4A, ABCB1 and ABCC4* which encode H19 lncRNA, CUL4A protein, MDR1 and MRP4 respectively integrate together in the molecular network to regulate chemoresistance is an intriguing challenge. As MDR1 is the major drug efflux factor, we explored the relationship of H19, CUL4A and MDR1 preliminarily by using RNA interference approach in Dox-resistant cells in the current study. First, we revealed that knockdown of *H19* in Dox-resistant cells resulted in a decrease in both *CUL4A* and *ABCB1*/MDR1 expression (Figures 4A and 5A), suggesting that H19 controls *CUL4* and *ABCB1* expression. Second, knockdown of *CUL4A* only caused a downregulation of *ABCB1*/MDR1 expression without any alteration in *H19* expression (Figure 5B), indicating that CUL4A regulates *ABCB1*/MDR1 expression and knockdown of CUL4A, which acts downstream of H19, blocks H19-induced ABCB1 expression. Finally, knockdown of *ABCB1* had no effect on both *H19* and *CUL4A* expression (Figures 4 and 5), suggesting it is located at the far downstream of this pathway. Further evidence to support that H19 and CUL4A are involved in the same molecular pathway comes from the analysis of gene expression profile and KEGG pathways in H19 and CUL4A knockdown breast cancer cells, in which seven molecular pathways affected by *CUL4A* knockdown in breast cancer cells are converged to those with *H19* knockdown (Table 3). These results collectively indicate that H19 is at the far upstream of this *H19*-*CUL4*-*ABCB1*/MDR1 pathway that play a critical role in multidrug resistance. Further analysis of this pathway is currently ongoing in the lab.

In summary, aiming to further understanding of molecular mechanisms of chemoresistance in breast cancer, we have demonstrated in the present study that H19 lncRNA was significantly upregulated in Dox-resistant breast cancer cells, which contributed to the cell resistance of multiple chemotherapeutic agents. Although the underlying molecular mechanism related to H19 upregulation and H19 control of target gene expression remains to be elucidated, we have revealed that H19-medidated chemoresistance is associated to the upregulation of *CUL4A* and *ABCB1*/MDR1 expression. Furthermore, using RNA interference analysis, we have elucidated for the first time that H19 lncRNA functions at the far upstream of the *H19*-*CUL4A*-*ABCB1*/MDR1 pathway, indicating that a drug-induced epigenetic alteration is an upstream event leading MDR1-mediated multidrug resistance. Thus, a demonstration of H19 lncRNA function and an identification of *H19*-*CUL4A*-*ABCB1*/MDR1 pathway in chemoresistance have shed new light to the understanding of chemoresistance and will provide new therapeutic targets and strategies for clinical management of breast cancer patients.

## MATERIALS AND METHODS

### Reagents

Doxorubicin (Dox) and cisplatin (CDDP) were purchased from Sigma-Aldrich (St. Louis, MO, USA), and dissolved in DMSO at a stock concentration of 100 mM and 50 mM, respectively. Epirubicin, pirarubicin and paclitaxel were purchased from Selleckchem (Houston, TX, USA), and dissolved in DMSO at a stock concentration of 100 mM, 20 mM and 50 mM, respectively.

### Cell lines

The human breast cancer cell line MCF-7 was purchased from American Type Culture Collection (ATCC, Rockefeller, Maryland, USA) and the Dox-resistant MCF-7 cells were established by exposing MCF-7 cells to incrementally increasing concentrations of doxorubicin. Briefly, MCF-7 cells were grown in RPMI-1640 medium (Gibco, BRL Co.Ltd., USA) supplemented with 10% fetal bovine serum (FBS), 20 mM L-glutamine, 100 U/ml penicillin and 100 μg/ml streptomycin (Gibco, BRL Co.Ltd., USA) in a 5% CO2–95% air humidified atmosphere at 37°C. The cells were treated with doxorubicin at an initial dose of 100 nM. When cells reached 80% confluence, the cells were passaged and advanced as a population by a 2-fold stepwise increase of the Dox concentration. The three Dox-resistant cell lines, MCF-7/Dox400, MCF-7/Dox800 and MCF-7/Dox1600, were obtained when cells were treated with Dox at concentrations of 400, 800 and 1600 nM, respectively. Three parallel control cell lines (MCF-7/Con400, MCF-7/Con800 and MCF-7/Con1600) that were treated with DMSO vehicle control were also obtained. The Dox-resistant and the parallel control cells were maintained in the same RPMI-1640 medium plus the corresponding concentration of Dox (400, 800, or 1600 nM) or DMSO (0.1%), respectively. Doxorubicin and DMSO were removed from Dox-resistant cells and parallel control cells 7 days before experiments, and all cells were cultured in phenol red-free RPMI-1640 medium supplemented with 5% charcoal-dextran-treated FBS (BI Technologies, Fullerton, CA, USA), 20 mM L-glutamine, 100 U/ml of penicillin and 100 μg/ml of streptomycin two days before experiments.

### Cell viability assay

To determine viable cell numbers, cells were plated in 96-well plates (5000 cells/well) and treated with various drugs as indicated for 48 h after cell attachment. Viable cell numbers were determined using a CellTiter AQueous One Solution Cell Proliferation Assay kit following the manufacturer's instructions (Promega, Madison, WI, USA). The data was expressed as percentage of the corresponding control. The 50% inhibition (IC_50_) of cell growth was determined by dose-response study and calculated relative to corresponding controls.

### Cell transfection and knockdown of gene expression by RNA interference

Synthetic RNA oligonucleotides targeting *H19, CUL4A* and *ABCB1* were obtained from RiboBio Co., Ltd. (Guangzhou, China). H19 lncRNA was knocked down using a specific H19 hairpin siRNA vector (shRNAH19). The H19 hairpin siRNA sequences are: 5′-CATCAAAGACACCATCGGA-3′ that were subcloned into pSilencer 2.1-U6 neovector (shRNAH19) (RiboBio Co., Ltd.) [21]. The sequences of *CUL4A* siRNA were 5′-GAAGCTGGTCATCAAGAAC-3′, and the sequences of *ABCB1* siRNA are 5′-GAAAC CAACTGTCAGTGTA-3′. A negative control shRNA and a negative control siRNA were also purchased from RiboBio.

Cell transfection was carried out as previously described [33]. Briefly, cells were plated in phenol red-free medium containing 5% stripped FBS in 12-well plates at a density of ∼50%. The transfections of various doses of siRNAs or shRNA were performed using Lipofectamine RNAiMAX or Lipofectamine 2000 reagent (Gibco, BRL Co.Ltd., USA) following the manufacturer's instructions. Twenty-four hours (h) after transfection, the cells were treated with various chemotherapeutic agents for different times and analyzed as indicated in each experiment.

To generate a stable H19 lncRNA knockdown cell line, H19 lncRNA shRNA was cloned into pRNAT-U6.1/Neo vector, and transfected to MCF7/Dox1600 cells using Lipofectamine 2000 as described above. The transfected cells were selected using G418 (Promega, Madison, WI, USA) and a stable clone was obtained and verified for H19 expression.

### Microarray analysis

Total cellular RNAs were extracted from various cell lines and quantified by the NanoDrop ND-2000 (Thermo Scientific, Massachusetts, USA). The RNA integrity was assessed using Agilent Bioanalyzer 2100 (Agilent Technologies, California PaloAlto, USA). The microarray was performed by Agilent Human lncRNA Microarray V5. Briefly, sample labeling, microarray hybridization and washing were performed based on the manufacturer's standard protocols (Agilent Technologies, California Palo Alto, USA). Total RNA was reverse-transcribed to cDNA, then synthesized into cRNA and labeled with Cyanine-3-CTP. The labeled cRNAs were hybridized onto the microarray. After washing, the arrays were scanned by the Agilent Scanner G2505C (Agilent Technologies). Feature Extraction software (version10.7.1.1, Agilent Technologies) was used to obtain an array of raw data. Genespring (version 13.1, Agilent Technologies) was employed to finish the basic analysis of the raw data, which was normalized with the quantile algorithm. The probes that had flags in “P” at least 1 condition out of 2 conditions were chosen for further data analysis. Differentially expressed genes or lncRNAs were then identified through fold changes as well as *P* values calculated with *t*-test. The threshold set for up- and down-regulated genes was a fold change ≥ 2.0 and a *P* value < 0.05. Afterwards, Gene Ontology (GO) analysis and Kyoto Encyclopedia of Genes and Genomes (KEGG) analysis were applied to determine the functional significances of these differentially expressed mRNAs. Finally, hierarchical clustering was performed to display the distinguishable gene expression pattern among samples.

### Quantitative real-time RT-PCR

Total RNA was reversely transcribed in a total volume of 20 μl with 200 units of reverse transcriptase, 50 pmol random hexamer and 1 mM deoxynucleotide triphosphates. The reaction products were then diluted to a total volume of 100 μl with distilled water. The real-time PCR reaction consisted of 2 μl of diluted reverse transcription product, 2X SYBR-Green Master Mix (Applied Biosystems, Foster City, CA, USA) and 0.3 μM forward and reverse primers. The primer pairs used were as follows: *H19*, 5′-GTCCGGCCTTCCTGAACACCTT-3′ and 5′-GCTTCACCTTCCAGAGCCGAT-3′; *CUL4A*, 5′-T GAGCGGTTCGTCAACCTG-3′ and 5′-ACATGCTTTGC GATCAGTTCTG-3′; *ABCB1*, 5′-CACCAGCATCATGAG AGGAAGTC-3′ and 5′-AAATTGGCTTGACAAGTTGTA TATGG-3′; β-*actin*, 5′-TTGATTTTGGAGGGATCTCG CTC-3′ and 5′-GAGTCAACGGATTTGGTCGTATTG-3′. The reaction was carried out in a Roche LightCycler 480 Sequence Detection System for 40 cycles (95°C for 15 seconds, 60°C for 1 min) after an initial 10-min incubation at 95°C. β-actin was used as an internal control. The level of RNA was expressed as fold of corresponding control calculated using the ΔΔCt method [33].

### Western blot analysis

Protein extraction and Western blot analysis were performed as previously described with minor modifications [33]. Briefly, total cellular proteins were extracted from the harvested cells using a lysis buffer [62.5 mM Tris-HCl pH 6.8, 100 mM dithiothreitol (DTT), 2% SDS and 10% glycerol]. The protein concentrations were determined using the Bicinchonininc acid method with the Bio-Rad protein assay following the manufacturer's instructions (Bio-Rad, Hercules, CA, USA). Cellular proteins were separated on sodium dodecyl sulfate polyacrylamide gels and transferred to polyvinylidene fluoride membranes. Blots were incubated in blocking buffer (5% non-fat dry milk in Tris-buffered saline with 0.5% Tween, TBS-T) at room temperature for 2 h. After washing with TBS-T, the nitrocellulose was incubated with a specific antibody against MDR1 (ab170904, Abcam, CA, USA), β-actin (AC-15, Sigma Chemical Co., St. Louis, MO, USA), MRP4 (#12705, Cell Signaling, CA, USA), XPD (ab167418, Abcam, CA, USA), TOPIIA (ab52934, Abcam, CA, USA), or CUL4A (ab92554, Abcam, CA, USA) overnight at 4°C. Following the incubation with a horseradish peroxidase-conjugated secondary antibody, the signal was detected using an ECL Western Blotting System (Promega, Madison, WI, USA) and visualized and quantitated using the Bio-Rad ChemiDoc MP system.

### Analysis of public breast cancer datasets

GEO2R, is an R-based web application that helps users analyse GEO data [42]. Public breast cancer microarray datasets were accessed and breast tumor expression data of *CUL4A* was downloaded (GSE52544). The “R scripts generated” data with *p* < 0.05 in GEO2R analysis of the GEO database were retained. All data were displayed as log2 transformed. (https://www.ncbi.nlm.nih.gov/geo/geo2r/?acc=GSE76540).

The Kaplan-Meier plotter is capable of assessing the effect of 54,675 genes on survival using 10,293 cancer samples based on a meta-analysis of biomarkers [43]. Kaplan-Meier plot, the hazard ratio (HR) with 95% confidence intervals and log rank *P* value were calculated and plotted in R using the “surplus” function of the “survival” Bioconductor package. The entire computational pathway was made accessible for reanalysis in platform independent online software running on a Debian Linux (http://www.debian.org) server powered by Apache (http://www.apache.org). The scripts on the server-side were developed in PHP, which control the user interface, the requests and the delivery of the results. The RODBC package provides a middleware layer between R and the PostgreSQL database. This platform can be reached over the internet via http://www.kmplot.com/breast cancer [44].

### Statistical analysis

Each experiment was performed at least three times, and the data are presented as mean ± SEM. For parametric data, One-way ANOVA following *post-hoc* Student-Newman-Keuls test was used to compare the difference among multiple groups, and the Student's *t*-test was used to determine the statistical significance between two groups using the SPSS software. A *p*-value less than 0.05 was considered to be statistically significant.

## SUPPLEMENTARY MATERIALS TABLE




