# Supplementary material for: LncRNA H19 is a major mediator of doxorubicin chemoresistance in breast cancer cells through a cullin4A-MDR1 pathway
Source: Oncotarget. 2017 Sep 21;8(54):91990–2003. doi: 10.18632/oncotarget.21121 (PMC5696158; doi:10.18632/oncotarget.21121)
Supplement: Supplementary file 1 [file oncotarget-08-91990-s001.pdf]

LncRNA H19 is a major mediator of doxorubicin chemoresistance in breast cancer cells through a cullin4A-MDR1 pathway

SUPPLEMENTARY MATERIALS

Supplementary Table 1: The differentially genes in MCF-7/Dox1600 vs parallel control MCF-7/Con1600 and MCF-7/DOX/shH19 VS MCF-7/DOX/NC. See Supplementary_Table_1
